# Application of a count regression model to identify the risk factors of under-five child morbidity in Bangladesh

**DOI:** 10.1093/inthealth/ihad107

**Published:** 2023-11-16

**Authors:** Md Ismail Hossain, Abu Sayed Md Ripon Rouf, Md Rukonozzaman Rukon, Shuvongkar Sarkar, Iqramul Haq, Md Jakaria Habib, Faozia Afia Zinia, Tanha Akther Tithy, Asiqul Islam, Md Amit Hasan, Mir Moshiur, Md Shakil Ahmed Hisbullah

**Affiliations:** Department of Statistics, Jagannath University, Dhaka-1100, Bangladesh; Department of Mathematics and Natural Sciences, BRAC University, Dhaka-1212, Bangladesh; Department of Statistics, Jagannath University, Dhaka-1100, Bangladesh; Department of Statistics, Jagannath University, Dhaka-1100, Bangladesh; Department of Statistics, Jagannath University, Dhaka-1100, Bangladesh; Department of Agricultural Statistics, Sher-e-Bangla Agricultural University, Dhaka-1207, Bangladesh; Department of Statistics, Jagannath University, Dhaka-1100, Bangladesh; Department of Statistics, Jagannath University, Dhaka-1100, Bangladesh; Department of Statistics, Jagannath University, Dhaka-1100, Bangladesh; Department of Statistics, Jagannath University, Dhaka-1100, Bangladesh; Department of Statistics, Jagannath University, Dhaka-1100, Bangladesh; Department of Statistics, Jagannath University, Dhaka-1100, Bangladesh; Department of Statistics, Jagannath University, Dhaka-1100, Bangladesh

**Keywords:** ARI, child health, child nutrition, diarrhoea, fever, under-five morbidity

## Abstract

**Background:**

Bangladesh has seen a significant decline in child mortality in recent decades, but morbidity among children <5 y of age remains high. The aim of this analysis was to examine trends and identify risk factors related to child morbidity in Bangladesh.

**Methods:**

This analysis is based on data from four successive cross-sectional Bangladesh Demographic and Health Surveys for the years 2007, 2011, 2014 and 2017–18. Several count regression models were fitted and the best model was used to identify risk factors associated with morbidity in children <5 y of age.

**Results:**

According to the results of the trend analysis, the prevalence of non-symptomatic children increased and the prevalence of fever, diarrhoea and acute respiratory infections (ARIs) decreased over the years. The Vuong's non-nested test indicated that Poisson regression could be used as the best model. From the results of the Poisson regression model, child age, sex, underweight, wasted, stunting, maternal education, wealth status, religion and region were the important determinants associated with the risk of child morbidity. The risk was considerably higher among women with a primary education compared with women with a secondary or greater education in Bangladesh.

**Conclusions:**

This analysis concluded that child morbidity is still a major public health problem for Bangladesh. Thus it is important to take the necessary measures to reduce child morbidity (particularly fever, diarrhoea and ARI) by improving significant influencing factors.

## Introduction

Morbidity refers to the condition of having symptoms or being in an unhealthy state due to a disease or medical condition. The World Health Organization (WHO) and United Nations Children's Fund (UNICEF) are collaboratively working together to tackle the five health challenges—pneumonia, diarrhoea, measles, malaria and malnutrition—that stand as the leading causes of child mortality, accounting for approximately two-thirds of child deaths in developing countries.^1^ Child health risks include low birthweight, malnutrition, lack of breastfeeding, overcrowding, access to unsafe food and drinking water and poor hygiene practices. Before delivery, a mother can increase her child's chances of survival and health by attending prenatal care, protecting against tetanus, getting vaccinated and avoiding smoking and alcohol consumption. In essence, the early detection and treatment of illnesses in infants and children are pivotal in improving a child's chances of survival.

As previously mentioned, child morbidity remains a leading contributor to child mortality. Despite the global under-five mortality rate decreasing by >55% during the past 2 decades, this reduction rate remains insufficient, particularly in low- and middle-income countries (LMICs).^2,3^ Many LMICs, including South Africa and Bangladesh, have yet to attain Sustainable Development Goal 3, despite their efforts to reduce mortality and morbidity. In 2020, sub-Saharan Africa (SSA) and South Asia jointly accounted for >80% of global deaths among children <5 y of age.^4^

In Bangladesh, 45 per 1000 live births died before their fifth birthday, and the majority of these deaths occurred from preventable and treatable causes.^5^ As in several LMICs, morbidity in children <5 y of age is widespread in Bangladesh, where diarrhoea, fever and acute respiratory infections (ARIs) were the most common childhood illnesses. Every year in Bangladesh, pneumonia accounts for approximately 19% of the total under-five deaths.^6^ Another study conducted in Bangladesh suggests that >20% of children <5 y of age suffer from colds and fever and approximately 17% from diarrhoea.^1^ Another common childhood illness treated as a global public health burden is fever.^7^

Numerous recent studies have sought to identify the risk factors associated with morbidity in children <5 y of age, often employing a widely used binary logistic regression model.^8–10^ For instance, a recent study conducted by Rahman and Hossain endeavoured to uncover the risk factors for diarrhoea, ARIs and fever in children by applying a binary logistic regression model, utilizing nationally representative survey data.^11^ Other studies have indicated that common risk factors influencing child morbidity encompass aspects such as sex and age,^12^ nutritional status (underweight, wasting, stunting),^13^ birth order,^14^ type of birth, maternal education,^14^ age at first birth, religion,^15^ wealth status,^16^ mass media,^17^ residence^13^ and geographical divisions (region)^13^ within a given country. However, the morbidity status of children can be classified as suffering from at least one disease, at least two diseases, at least three diseases and so on. By considering this counting nature child morbidity status, one can apply various count regression models to determine the risk factors for child morbidity. In this context, this study used various count regression models, namely Poisson regression, negative binomial regression, zero-inflated Poisson regression, etc, which may provide a better solution when it satisfies the count regression assumption. However, to the authors’ knowledge, no results are available to identify the level and trends in the prevalence and symptoms of fever, diarrhoea and ARIs in Bangladesh. The objectives of this study were made keeping in mind these research gaps, including exploring the levels and trends of the prevalence of under-five child morbidity and examining the influence of demographic factors on child morbidity and identifying the risk factors from the best count regression model using data from four successive Bangladesh Demographic and Health Surveys (BDHSs).

## Methods

### Source of data

Survey data from four successive BDHSs (2007, 2011, 2014 and 2017–2018) were used in this study. The BDHS is a national representative survey dataset that is financially supported by the US Agency for International Development (USAID) in Bangladesh.

### Sample design and sample size

A two-stage stratified sampling procedure was applied in the BDHS, where the enumeration areas (EAs) were selected in the first stage and a systematic sample of households per EA was selected in the second stage. The BDHSs covered 10 819, 18 000, 18 000 and 20 250 residential households in 2007, 2011, 2014 and 2017–2018, respectively. From these sampled households, 5719, 8395, 7760 and 8421 mothers/caretakers with children <5 y of age (who were alive) were interviewed, respectively.

### Dependent variable

Child morbidity tracks the occurrence of illnesses, diseases or health problems in children within a certain population and time frame, indicating the frequency of their health challenges. The BDHS provided three types of childhood diseases, specifically focusing on the information from the 2 weeks leading up to the survey. These are fever, diarrhoea and ARI.

Another recent study conducted in Bangladesh also considered these three diseases and categorized them as part of child morbidity.^13^ For this study, we define our dependent variable as follows: if the child experienced at least one problem, then this study numbered it as ‘1’; if the child experienced at least two problems, then this study numbered it as ‘2’; if the child experienced all of these problems, then this study numbered it as ‘3’; and if the child experienced none of these problems, then this study numbered it as ‘0’. That is, the outcome variable for this study consists of non-negative integer values (0–3).

### Independent variable

A set of sociodemographic risk factors was considered that can determine the morbidity of children <5 y of age in Bangladesh. In this study, child age in months (<12, 12–35, 36–59), child sex (male, female), underweight (yes, no), wasted (yes, no), stunted (yes, no), birth order (1, 2–3, >3), type of birth (single, multiple), maternal education (none, primary, secondary+), religion (Muslim, other), wealth status (poor, middle, rich), mass media access (yes, no), residence (rural, urban) and region (northern, southern, central, eastern) were selected as independent variables. The BDHS dataset encompasses data from eight primary administrative divisions in Bangladesh, namely Barishal, Chattogram, Dhaka, Khulna, Rajshahi, Rangpur, Mymensingh and Sylhet. To simplify the analysis by geographic location, we grouped Rajshahi and Rangpur into the northern region, Chattogram and Sylhet into the eastern region, Dhaka and Mymensingh into the central region and Barishal and Khulna into the southern region.

### Statistical analysis

#### Normality test

This study applied the Q-Q plot and the Kolmogorov–Smirnov test to justify the normality assumptions of the outcome variable.^18^ Since the number of health problems experienced by a child <5 y of age was a count response variable and all of the observations were greater than or equal to zero, it is important to test the normality assumption.

#### Count regression models

Count data analysis involves several methods designed to address non-normality in dependent variables. This study focuses on four key models: Poisson regression, negative binomial (NB) regression, zero-inflated Poisson (ZIP) modelling and zero-inflated negative binomial (ZINB) modelling. Now let's delve into the specific details about these models.

The Poisson regression model, a basic count model, assumes equality between the variance and mean of the count variable and independence of occurrence.^19^ According to this study environment, let ${Y}_1,{Y}_2, \ldots ,{Y}_n$ be the $n$ independent random variables, where ${y}_i$ denotes the total number of health problems of the ${i}^{th}$ child. Then the Poisson equation of ${Y}_i$ with rate parameter ${\lambda }_i$ can be defined as follows:


\begin{eqnarray*}
P\left[ {{Y}_i = {y}_i} \right] = \frac{{{e}^{ - {\lambda }_i\ }\lambda _i^{{y}_i}}}{{{y}_i!}}.
\end{eqnarray*}

Here, ${\lambda }_i > 0$ and ${y}_i = 0,1,2, \ldots ,\infty $.

In practice, count data often exhibit overdispersion, where the variance exceeds the mean.^20^ In such cases, the NB model, accommodating dispersion through an additional heterogeneity parameter, offers a suitable alternative.^21,22^ Mathematically the NB model can be written as follows:


\begin{eqnarray*}P\left[ {{Y}_i = {y}_i} \right] = \frac{{\Gamma \left( {{y}_i + \frac{1}{\alpha }} \right)\ }}{{\Gamma \left( {{y}_i + 1} \right)\ \Gamma \left( {\frac{1}{\alpha }} \right)}}\ {\left( {\frac{{\alpha {\lambda }_i}}{{1 + \alpha {\lambda }_i}}} \right)}^{{y}_i}\ {\left( {\frac{1}{{1 + \alpha {\lambda }_i}}} \right)}^{\frac{1}{\alpha }},\end{eqnarray*}

where $\alpha \ge 0$ served as the dispersion parameter and ${y}_i = 0,1,2, \ldots $.

Count data often contain excessive zeros, which can affect traditional Poisson or NB models. To address this, the study considered the ZIP and ZINB models introduced by Lambert^23^ and Heilbron,^24^ respectively. Therefore, the ZIP model probability mass function can be written as


\begin{eqnarray*}
P\left[ {{Y}_i = {y}_i} \right] = \left\{ {\begin{array}{@{}l@{\quad }l@{}}
\phi + \left( {1 - \phi } \right){e}^{ - {\lambda }_i\ },& if\ {y}_i = 0\\
\left( {1 - \phi } \right)\frac{{{e}^{ - {\lambda }_i\ }\lambda _i^{{y}_i}}}{{{y}_i!}}, & if\ {y}_i = 1,2 \end{array}} \right..
\end{eqnarray*}

Here, $0 \le \phi < 1,$ and the model incorporates extra zeros compared with the original Poisson model when ($\phi = 0$).

For the ZINB distribution, the function is given by


\begin{eqnarray*}
P\left[ {{Y}_i = {y}_i} \right] = \left\{ {\begin{array}{@{}l@{\quad }l@{}} \phi + \left( {1 - \phi } \right)\ {{\left( {1 + \alpha {\lambda }_i} \right)}}^{ - \frac{1}{\alpha }},& if\ {y}_i = 0\\
\left( {1 - \phi } \right)\frac{{\Gamma \left( {{y}_i + \frac{1}{\alpha }} \right)}}{{\Gamma \left( {{y}_i + 1} \right)\ \Gamma \left( {\frac{1}{\alpha }} \right)}}\ {{\left( {\frac{{\alpha {\lambda }_i}}{{1 + \alpha {\lambda }_i}}} \right)}}^{{y}_i}\ {{\left( {\frac{1}{{1 + \alpha {\lambda }_i}}} \right)}}^{\frac{1}{\alpha }},& if\ {y}_i > 0 \end{array}} \right.\end{eqnarray*}

where the parameters ${\lambda }_i\!\!{}$ and $\phi{}\!\! $ depend on the covariates and $\alpha \ge 0$ is a scalar, indicating overdispersion when either $ \!{}\phi{}\! $ or $\alpha $ is >0. Thus this equation reduces to NB when $\phi = 0$ and to the ZIP when $\alpha = 0. $

#### The Vuong closeness test

In this study, we employed the Vuong closeness test, a well-established model selection technique. This test is based on likelihood ratios and uses the Kullback–Leibler information criterion to evaluate nested, non-nested or overlapping models.^25^ According to Vuong,^25^ the null hypothesis was that competing models are equally close to the true data generation process compared with the alternative hypothesis that one model is closer.

### Analytical software

The Statistical Package for Social Sciences version 25 (IBM, Armonk, NY, USA) was used for data wrangling and R version 4.0 (R Foundation for Statistical Computing, Vienna, Austria) was used for the analysis.

### Ethical approval

This study utilized a publicly accessible secondary dataset obtained from the Demographic and Health Surveys (DHS) Program website (https://dhsprogram.com/data/). As the dataset was pre-existing and did not involve primary data collection, no additional ethics approval was necessary for its use.

## Results

### Trends in the prevalence of child morbidity

**Figure 1.**
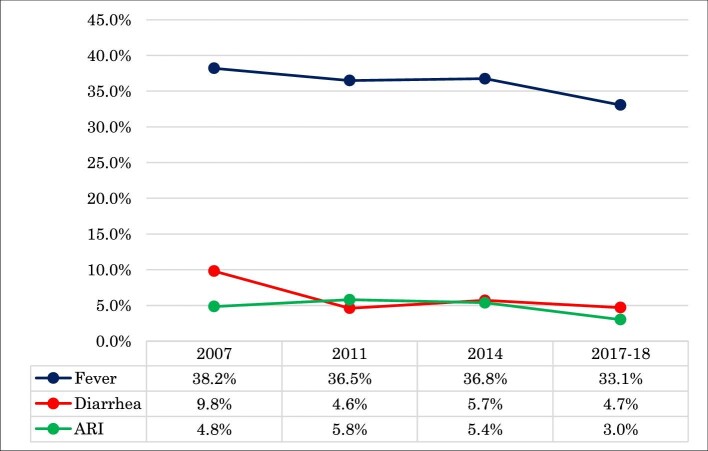
Trends in prevalence and symptoms of fever, diarrhoea and ARIs.

Figure 1 shows that 38.2%, 36.5%, 36.8% and 33.1% of children <5 y of age were fevered in 2007, 2011, 2014 and 2017–2018, respectively. The prevalence of diarrhoea was 9.8%, 4.6%, 5.7% and 4.7% among children <5 y of age in 2007, 2011, 2014 and 2017–2018, respectively. Among the survey years, 2011 had the highest percentage of children (5.8%) who suffered from ARI.

**Figure 2.**
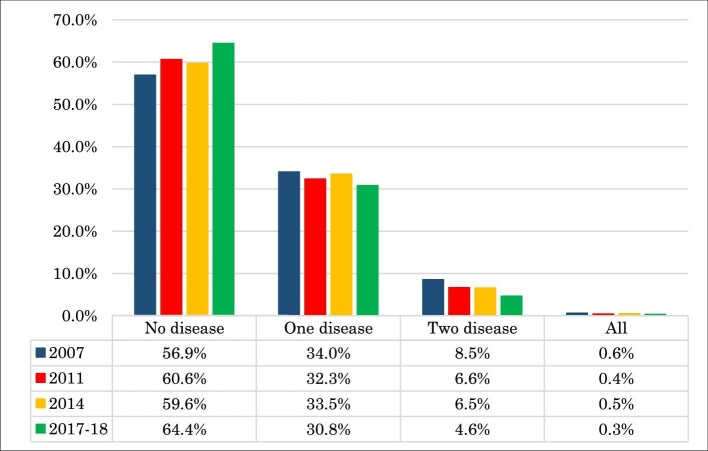
Prevalence of childhood illnesses by year.

Figure 2 demonstrates the trends in the prevalence of childhood illness. From this we see that the rate of the prevalence of no disease in 2007 was 56.9%, which increased to 64.4% in 2017–2018, with rates of 60.6% in 2011 and 59.6% in 2014. The prevalence of at least one disease was 34.0% in 2007, 32.3% in 2011, 33.5% in 2014 and 30.8% in 2017–2018. In 2007 the rate of having two diseases was 8.5% and in 2017–2018 it was 4.6%. The prevalence rate of all diseases combined was 0.6% in 2007, 0.4% in 2011, 0.5% in 2014 and 0.3% in 2017–2018.

### Normality assumption test

**Figure 3.**
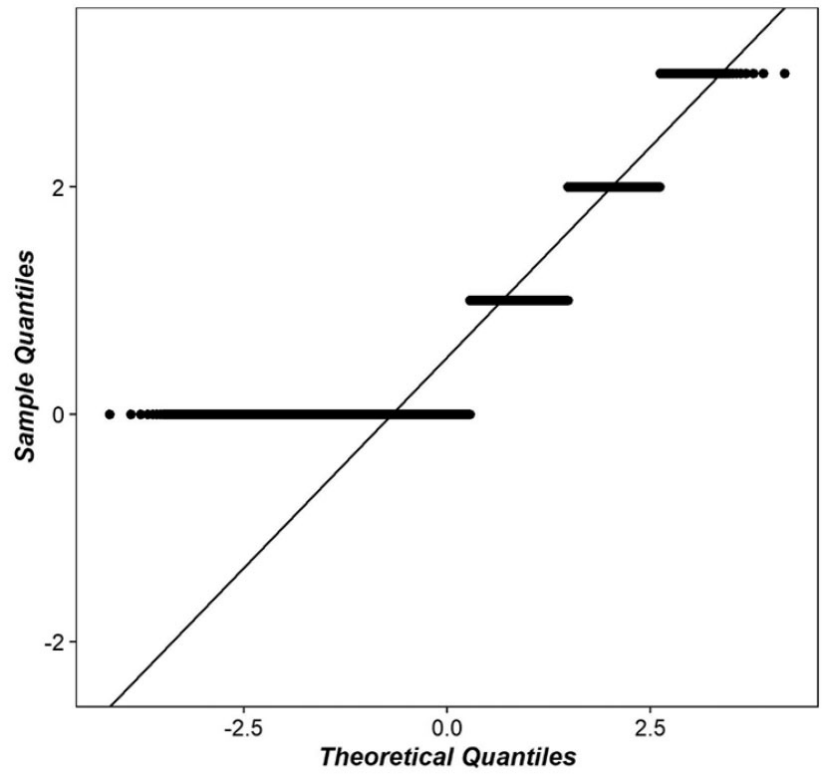
Normality test by the Q-Q plot technique.

Figure 3 represents a Q-Q plot where we see that the points deviate significantly from the straight diagonal line, indicating that the set of data is not normally distributed. For another indication of normality, this study also used the Kolmogorov–Smirnov normality test, where the p-value is <0.001, which strongly suggests that the data are not normally distributed.

### Background characteristics of respondents

Table 1 illustrates the frequency distribution of respondents according to their socio-economic and demographic characteristics. Table 1 shows that the highest percentage of children's age across the study was 36–59 months (40.4%) followed by 12–35 months (39.5%) and <12 months (20.1%). It shows that the number of male (51.3%) and female children (48.7%) was almost equal. Table 1 shows the proportion of variables was higher among children who were not underweight (67.9%), not wasted (86.4%) and not stunted (62.7%). Moreover, Table 1 revealed that 16.9% of the mothers had no education and 29.5% did not finish primary school, while approximately half of the mothers (53.6%) completed secondary education or higher. Most of the mother's (74.1%) were <20 y of age at the time of their first delivery and 91.7% of the children belong to the Muslim religion. It further shows that 42.6% of children are from poor households, 19.2% are from middle-class households and 38.2% are from rich households. Approximately half of the mothers (51.6%) had access to the media. Most of the children (75.7%) were from rural areas. Almost one-third of children (33.1%) were from the central region, 30.4% were from the eastern region, 21.9% were from the northern region and 14.5% were from the southern region. The highest percentage of children in this study were from the survey year 2017–2018 (27.8%) followed by 2011 (27.7%), 2014 (25.6%) and 2007 (18.9%).

**Table 1.** Percentage distribution of respondents by socio-economic and demographic characteristics

Variables	Frequency	Percentage
Child age (months)		
<12	6088	20.1
12–35	11 974	39.5
36–59	12 234	40.4
Child sex		
Male	15 555	51.3
Female	14 740	48.7
Underweight child		
No	20 579	67.9
Yes	9716	32.1
Wasted child		
No	26 163	86.4
Yes	4132	13.6
Stunted child		
No	19 006	62.7
Yes	11 289	37.3
Maternal education		
None	5109	16.9
Primary	8950	29.5
≥Secondary	16 236	53.6
Mother's age at first birth (years)		
<20	22 458	74.1
≥20	7837	25.9
Religion		
Muslim	27 786	91.7
Other	2509	8.3
Wealth status		
Poor	12 904	42.6
Middle	5817	19.2
Rich	11 573	38.2
Mass media access		
No	14 670	48.4
Yes	15 625	51.6
Residence		
Rural	22 937	75.7
Urban	7358	24.3
Region		
Northern	6641	21.9
Southern	4406	14.5
Central	10 028	33.1
Eastern	9219	30.4
Survey year		
2007	5719	18.9
2011	8395	27.7
2014	7760	25.6
2017–2018	8421	27.8

### Multicollinearity diagnostics

Table 2 presents the correlation between independent variables, aiming to detect multicollinearity, which arises when two or more independent variables in the regression model are highly correlated. The presence of multicollinearity can be assessed using the tolerance and its reciprocal, known as the variance inflation factor (VIF). Multicollinearity is considered absent when each variable's VIF is <5 and the tolerance value for each variable is <0.1. Based on the analysis, it can be concluded that multicollinearity was not observed in this study. The VIF values ranged from 1.003 to 1.283.

**Table 2.** Collinearity diagnostics among the independent variables

	Collinearity statistics	
Variables	Tolerance	VIF
Child age	0.970	1.031
Child sex	0.997	1.003
Underweight child	0.779	1.283
Wasted child	0.794	1.259
Stunted child	0.782	1.279
Maternal education	0.783	1.277
Age at first birth	0.947	1.056
Religion	0.989	1.012
Wealth status	0.964	1.037
Mass media	0.890	1.124
Residence	0.815	1.226
Region	0.957	1.045
Survey year	0.936	1.068

### Model selection based on the Vuong non-nested test

Table 3 compares the models on the basis of Vuong non-nested test. All the models were compared by the Akaike information criterion (AIC), Bayes information criterion (BIC) and Raw tests. When the Poisson regression model and negative binomial regression (NBR) model were compared by Raw, AIC and BIC tests, the Poisson regression model was found to be significantly more preferable than the NBR (p<0.001). The Poisson regression model was also preferable to zero-inflated binomial regression (ZIBR) model in AIC and BIC tests, while the Raw test was not significant (p>0.05). Finally, when the Poisson regression model was compared with the zero-inflated negative binomial regression (ZINBR) model, the Poisson regression model was found to be preferable according to all three tests. Here, in both the AIC and BIC test, the Poisson regression model was significantly preferable to the ZINBR (p<0.001) while the raw test is not significant (p>0.05). The comparison showed that the Poisson regression model was approximately more preferable compared with the NBR, ZIBR and ZINBR models. Thus the Poisson regression model could be used as the best model.

**Table 3.** Vuong non-nested test results

		Vuong test		
Compare models	Test name	p-Value	Decision for superior	Preferable model
PR vs NBR	Raw	<0.001	PR>NBR	PR
	AIC	<0.001	PR>NBR	PR
	BIC	<0.001	PR>NBR	PR
PR vs ZIBR	Raw	0.46	PR<ZIBR	ZIBR
	AIC	<0.001	PR>ZIBR	PR
	BIC	<0.001	PR>ZIBR	PR
PR vs ZINBR	Raw	0.48	PR>ZINBR	PR
	AIC	<0.001	PR>ZINBR	PR
	BIC	<0.001	PR>ZINBR	PR

### Identify risk factors contributing to child morbidity

Table 4 presents the outcomes of the Poisson regression model used for estimating the parameters associated with child morbidity. Within this well-fitted model, the residual deviance serves as a valuable metric for conducting an overall goodness-of-fit assessment, indicating the model's strong alignment with the provided data. Children <12 months of age had 1.44 times (95% confidence interval [CI] 1.38 to 1.51) more risk and children 12–35 months of age had 1.37 times (95% CI 1.32 to 1.42) more risk of suffering from morbidity than children 36–59 months of age. Male children had 1.07 times (95% CI 1.04 to 1.11) higher risk than female children. The underweight child, the wasted child and the stunted child had an 11% (95% CI 1.06 to 1.17), 13% (95% CI 1.07 to 1.19) and 5% (95% CI 1.01 to 1.10) higher risk of morbidity, respectively, than the normal child.

**Table 4.** Poisson regression model for parameter estimation of child morbidity

Variable	Relative risk	95% CI	p-Value
Child age (months)			
<12	1.44	1.38 to 1.51	**<0.001**
12–35	1.37	1.32 to 1.42	**<0.001**
36–59 (Ref.)	1		
Child sex			
Male	1.07	1.04 to 1.11	**<0.001**
Female (Ref.)	1		
Underweight child			
Yes	1.11	1.06 to 1.17	**<0.001**
No (Ref.)	1		
Wasted child			
Yes	1.13	1.07 to 1.19	**<0.001**
No (Ref.)	1		
Stunted child			
Yes	1.05	1.01 to 1.10	**0.02**
No (Ref.)	1		
Maternal education			
None	1.00	0.95 to 1.05	0.99
Primary	1.08	1.04 to 1.12	**<0.001**
≥Secondary (Ref.)	1		
Age at first birth			
<20	1.06	1.02 to 1.11	**0.002**
≥20 (Ref.)	1		
Religion			
Muslim	1.20	1.12 to 1.28	**<0.001**
Other (Ref.)	1		
Wealth status			
Poor	1.08	1.03 to 1.13	**0.003**
Middle	1.09	1.03 to 1.14	**0.001**
Rich (Ref.)	1		
Mass media access			
No	1.00	0.96 to 1.04	0.95
Yes (Ref.)	1		
Residence			
Rural	1.03	0.99 to 1.07	0.15
Urban (Ref.)	1		
Region			
Northern	0.96	0.91 to 1.00	**0.05**
Southern	0.93	0.89 to 0.97	**0.001**
Central	0.89	0.84 to 0.93	**<0.001**
Eastern (Ref.)	1		
Survey year			
2007	1.22	1.16 to 1.29	**<0.001**
2011	1.13	1.08 to 1.19	**<0.001**
2014	1.11	1.06 to 1.17	**<0.001**
2017–2018 (Ref.)	1		
Goodness-of-fit test			
Pseudo R^2^	0.237		
Residual deviance	26 979		
AIC	51 870		

Significant values in bold.

The child born to a mother with a primary education had an 8% (95% CI 1.04 to 1.12) higher risk of morbidity than mothers who attended secondary or higher education. Children whose mothers were <20 y of age at delivery had a 6% higher risk (95% CI 1.02 to 1.11) of developing any disease than children whose mothers were ≥20 y of age at delivery. Muslim children had a 20% higher risk (95% CI 1.12 to 1.28) of morbidity than children of other religions. Children of poor families had an 8% higher risk (95% CI 1.03 to 1.13) and those of middle families had a 9% higher risk (95% CI 1.03 to 1.14) of morbidity than children of rich families.

Children from the northern region had 0.96 times (95% CI 0.91 to 1.00), southern region had 0.93 times (95% CI 0.89 to 0.97) and central region had 0.89 times (95% CI 0.84 to 0.93) lower risk of suffering from morbidity than children from the eastern region. Results show that the risk of childhood morbidity was higher in previous years than in 2017–2018.

## Discussion

This study is based on secondary data from four consecutive BDHSs (2007, 2011, 2014 and 2017–2018) to investigate trends in under-five child morbidity and identify potential risk factors associated with child morbidity in Bangladesh. Various count regression models were employed to detect potential risk factors related to child morbidity. Before applying these models, the Voung test was conducted to determine the most suitable count regression model. The results of the Voung test favoured the Poisson regression model over other count regression models. Subsequently, the Poisson regression model revealed that all covariates, except mothers’ mass media access and their residence, were significant risk factors for child morbidity in Bangladesh.

Child age plays a crucial role in child morbidity, with children <12 months having a higher likelihood of experiencing morbidity compared with those 36–59 months of age. Our findings align with previous studies conducted in Bangladesh,^13,26^ SSA,^27^ Ethiopia^28,29^ and Ghana,^30^ all of which reported similar results. These studies consistently highlight that younger children face a significantly higher risk of morbidity compared with their older counterparts.

An intriguing finding emerged, indicating that male children exhibited greater odds of experiencing morbidity compared with their female counterparts. This result is in line with previous research conducted in Bangladesh^13,31^ and SSA,^27^ where it was also observed that male children have a higher risk of morbidity. However, it is worth noting that a different study conducted in Bangladesh reported the opposite result, suggesting that child sex has no significant impact on child morbidity.^26^

Regarding the status of child underweight, it was observed that underweight children had a higher likelihood to experience morbidity compared with children classified as normal weight when using the latter as a reference category. Another similar study conducted in Bangladesh using a machine learning logistic classifier approach showed that there was a statistically significant difference between childhood morbidity and underweight children in Bangladesh.^13^ Additionally, children who suffered from wasting exhibited greater odds of child morbidity compared with their counterparts. It should be noted that nutrition-related issues, including stunting, wasting and being underweight, contribute to approximately 45% of all child mortalities in the under-five age group.^32^

Children whose mothers completed primary education were more likely to suffer from child morbidity than children whose mothers completed secondary or above education. Children in SSA whose mothers completed secondary or higher education were less likely to have diarrhoea than children whose mothers had a primary school education or had no education at all.^27^ Conversely, another study did not find any significant difference between maternal education and child morbidity.^26^ In the different settings, Haq et al.^33^ found that children whose mothers had no formal education had a higher risk of mortality compared with those whose mothers had a secondary or higher education in both urban and rural areas of Bangladesh.

Our analysis found that children born to mothers <20 y of age at delivery had a higher risk of developing various diseases compared with children born to mothers who were ≥20 y of age at delivery. Compared with children of young and middle-aged women, children of older women had a lower risk of experiencing morbidity.^27,34,35^ Furthermore, religion was an important factor for child morbidity in this study. Children of Muslim mothers had a higher risk of morbidity than those of non-Muslim mothers. Compared with other religions, Muslims had lower immunization coverage for all their children.^36^ While one study did not directly investigate child morbidity, it provides additional data relevant to our research, indicating that Muslim women in rural areas experience a higher likelihood of child mortality compared with their non-Muslim counterparts.^33^

In the present analysis, economic status had a notable impact on morbidity among children <5 y of age. Children from middle-class families were more likely to have child morbidity than those from rich families, and this result was consistent with previous studies conducted in Bangladesh^13,31^ and Nigeria.^37,38^ A pooled analysis from 31 SSA countries also found similar results to our analysis and observed that children from rich households were less likely to suffer from morbidity than children from poor households.^27^ In a study of under-five mortality in Ethiopia, the household wealth index had no notable impact on child morbidity.^39^

Our findings highlight that the geographic region of the respondents plays a significant role in child morbidity in Bangladesh. Children residing in the northern, southern and central regions of the country exhibited a lower likelihood of experiencing morbidity compared with their counterparts in the eastern region. According to a study based on the 2011 BDHS, children in the Chattogram division had a higher risk of morbidity compared with those in the Dhaka division.^26^ Rural mothers in the Dhaka, Rajshahi, Rangpur and Sylhet divisions were more likely to experience a child's death than those in the Barisal division.^33^ Regarding the BDHSs, a negative correlation was observed between child morbidity and the survey year. Compared with the 2017–2018 BDHS, the likelihood of child morbidity was higher in all three preceding BDHSs conducted in Bangladesh.

This study endeavoured to elucidate patterns and linked risk factors contributing to child morbidity in Bangladesh. Despite its strengths, several limitations need acknowledgment. First, due to data constraints, this study could not incorporate several significant factors (mother's anaemia level, prenatal care, antenatal care, information regarding complications in pregnancy, size of the child at birth, immunization status etc.) that play a crucial role in the health of children <5 y of age. Second, the cross-sectional nature of the data prevents the establishment of causal relationships. Indeed, the BDHSs are cross-sectional in nature and do not provide specific seasonal data for individual years, as they do not collect data continuously over time. Consequently, it is challenging to assess seasonality trends based on this type of data, which primarily captures a snapshot of health indicators at specific points in time.

## Conclusions

From the perspective of this research, it can be said that child morbidity is still a major public health problem in Bangladesh. Therefore, the government of Bangladesh and policymakers should focus on the child health sector. In particular, they need to improve various factors, such as child nutrition, maternal education, wealth status etc., that significantly influence child morbidity. The media can reach the public in a short time, thus there is a need to increase media coverage of child healthcare in Bangladesh.

## Data Availability

Data in this study was from the BDHS, available from https://dhsprogram.com/data/.

## References

[1] Anne RA, Akhter N, Shapla NR et al. Pattern of morbidities in under five children and health seeking behaviour of their parents. J Armed Forces Med Coll Bangladesh. 2015;11(1):59–63.

[2] Sharrow D, Hug L, You D et al. Global, regional, and national trends in under-5 mortality between 1990 and 2019 with scenario-based projections until 2030: a systematic analysis by the UN Inter-agency Group for Child Mortality Estimation. Lancet Glob Health. 2022;10(2):e195–206. 35063111 10.1016/S2214-109X(21)00515-5 PMC8789561

[3] Tekin M . Under-five mortality causes and prevention. In: Bacha U, editor. Mortality rates in middle and low-income countries. IntechOpen; 2022. Available from: 10.5772/intechopen.100526

[4] World Health Organization . Child mortality (under 5 years). Available from: https://www.who.int/news-room/fact-sheets/detail/levels-and-trends-in-child-under-5-mortality-in-2020

[5] National Institute of Population Research and Training, ICF . Bangladesh Demographic and Health Survey 2017–18. Dhaka, Bangladesh, and Rockville, MD: NIPORT and ICF; 2020.

[6] Rahman AE, Hossain AT, Siddique AB et al. Child mortality in Bangladesh – why, when, where and how? A national survey-based analysis. J Glob Health. 2021;11:04052. 34552721 10.7189/jogh.11.04052 PMC8442576

[7] Kiemde F, Tahita MC, Lompo P et al. Treatable causes of fever among children under five years in a seasonal malaria transmission area in Burkina Faso. Infect Dis of Poverty. 2018;7(1):60. 29891004 10.1186/s40249-018-0442-3 PMC5994647

[8] Paul P, Mondal D. Maternal experience of intimate partner violence and its association with morbidity and mortality of children: evidence from India. PLoS One. 2020;15(4):e0232454. 32353037 10.1371/journal.pone.0232454 PMC7192445

[9] Ullah MB, Mridha MK, Arnold CD et al. Factors associated with diarrhea and acute respiratory infection in children under two years of age in rural Bangladesh. BMC Pediatr. 2019;19:386. 31656181 10.1186/s12887-019-1738-6 PMC6815354

[10] Savitha A, Gopalakrishnan S. Determinants of acute respiratory infections among under five children in a rural area of Tamil Nadu, India. J Fam Med Prim Care. 2018;7(6):1268–73. 10.4103/jfmpc.jfmpc_131_18 PMC6293935 30613509

[11] Rahman A, Hossain MM. Prevalence and determinants of fever, ARI and diarrhea among children aged 6–59 months in Bangladesh. BMC Pediatr. 2022;22:1–12. 35248016 10.1186/s12887-022-03166-9 PMC8897933

[12] Abdulkadir MB, Abdulkadir ZA. A cross-sectional survey of parental care-seeking behavior for febrile illness among under-five children in Nigeria. Alex J Med. 2017;53(1):85–91.

[13] Methun MIH, Kabir A, Islam S et al. A machine learning logistic classifier approach for identifying the determinants of under-5 child morbidity in Bangladesh. Clin Epidemiol Glob Health. 2021;12:100812.

[14] Adesanya O, Chiao C. Environmental risks associated with symptoms of acute respiratory infection among preschool children in north-western and south-southern Nigeria communities. Int J Environ Res Public Health. 2017;14(11):1396–405. 29144416 10.3390/ijerph14111396 PMC5708035

[15] Anteneh ZA, Andargie K, Tarekegn M. Prevalence and determinants of acute diarrhea among children younger than five years old in Jabithennan District, Northwest Ethiopia, 2014. BMC Public Health. 2017;17:99. 28103908 10.1186/s12889-017-4021-5 PMC5248477

[16] Lwin KS, Nomura S, Yoneoka D et al. Associations between parental socioeconomic position and health-seeking behaviour for diarrhoea and acute respiratory infection among under-5 children in Myanmar: a cross-sectional study. BMJ Open. 2020;10:e032039. 10.1136/bmjopen-2019-032039 PMC7170571 32220909

[17] Demissie GD, Yeshaw Y, Aleminew W et al. Diarrhea and associated factors among under five children in sub-Saharan Africa: evidence from demographic and health surveys of 34 sub-Saharan countries. PLoS One. 2021;16(9):e0257522. 34543347 10.1371/journal.pone.0257522 PMC8452002

[18] Ghasemi A, Zahediasl S. Normality tests for statistical analysis: a guide for non-statisticians. Int J Endocrinol Metab. 2012;10(2):486–9. 23843808 10.5812/ijem.3505 PMC3693611

[19] Lawless JF . Negative binomial and mixed Poisson regression. Can J Stat. 1987;15(3):209–25.

[20] Cameron AC, Trivedi PK. Regression analysis of count data. Cambridge: Cambridge University Press; 2013.

[21] Miaou S-P . The relationship between truck accidents and geometric design of road sections: Poisson versus negative binomial regressions. Accid Anal Prev. 1994;26(4):471–82. 7916855 10.1016/0001-4575(94)90038-8

[22] Hilbe J . Negative binomial regression. Cambridge: Cambridge University Press; 2011.

[23] Lambert D . Zero-inflated Poisson regression, with an application to defects in manufacturing. Technometrics. 1992;34(1):1–14.

[24] Heilbron DC . Zero-altered and other regression models for count data with added zeros. Biometr J. 1994;36(5):531–47.

[25] Vuong QH . Likelihood ratio tests for model selection and non-nested hypotheses. Econometrica. 1989;57(2):307–33.

[26] Kamal MM, Hasan MM, Davey R. Determinants of childhood morbidity in Bangladesh: evidence from the Demographic and Health Survey 2011. BMJ Open. 2015;5:e007538. 10.1136/bmjopen-2014-007538 PMC4636670 26510724

[27] Adedokun ST, Yaya S. Childhood morbidity and its determinants: evidence from 31 countries in sub-Saharan Africa. BMJ Glob Health. 2020;5:e003109. 10.1136/bmjgh-2020-003109 PMC7552796 33046457

[28] Takele K, Zewotir T, Ndanguza D. Risk factors of morbidity among children under age five in Ethiopia. BMC Public Health. 2019;19:942. 31307433 10.1186/s12889-019-7273-4 PMC6631490

[29] Mihrete TS, Alemie GA, Teferra AS. Determinants of childhood diarrhea among underfive children in Benishangul Gumuz Regional State, North West Ethiopia. BMC Pediatr. 2014;14:102. 24731601 10.1186/1471-2431-14-102 PMC4021233

[30] Kumi-Kyereme A, Amo-Adjei J. Household wealth, residential status and the incidence of diarrhoea among children under-five years in Ghana. J Epidemiol Glob Health. 2016;6(3):131. 26070430 10.1016/j.jegh.2015.05.001 PMC7320473

[31] Rahman M, Hosen A, Khan MA. Association between maternal high-risk fertility behavior and childhood morbidity in Bangladesh: a nationally representative cross-sectional survey. Am J Trop Med Hyg. 2019;101(4):929–36. 31333165 10.4269/ajtmh.19-0221 PMC6779183

[32] World Health Organization . Children: reducing mortality. Geneva: World Health Organization; 2020. Available from: https://www.who.int/news-room/fact-sheets/detail/children-reducing-mortality

[33] Haq I, Alam M, Islam A et al. Influence of sociodemographic factors on child mortality in Bangladesh: a multivariate analysis. J Public Health. 2020;30:1079–86.

[34] Vasconcelos MJdOB, Rissin A, Figueiroa JN et al. Factors associated with diarrhea in children under five years old in the State of Pernambuco, according to surveys conducted in 1997 and 2006. Rev Saúde Pública. 2018;52:48. 29723386 10.11606/S1518-8787.2018052016094 PMC5947442

[35] Gayawan E, Aladeniyi OB, Oladuti OM, et al. Investigating the spatial patterns of common childhood morbidity in six neighboring West African countries. J Epidemiol Glob Health. 2019;9(4):315–23. 31854175 10.2991/jegh.k.191030.001 PMC7310792

[36] Costa JC, Weber AM, Darmstadt GL et al. Religious affiliation and immunization coverage in 15 countries in sub-Saharan Africa. Vaccine. 2020;38(5):1160–9. 31791811 10.1016/j.vaccine.2019.11.024 PMC6995994

[37] Adesanya OA, Darboe A, Mendez Rojas B et al. Factors contributing to regional inequalities in acute respiratory infections symptoms among under-five children in Nigeria: a decomposition analysis. Int J Equity Health. 2017;16:16. 28784132 10.1186/s12939-017-0626-7 PMC5545834

[38] Nworie KM, Oyine Aluh D. Determinants of diarrhea and optimal childcare among under-five children in Nigeria: insights from the 2013 Demographic and Health survey. Fam Med Med Sci Res. 2018;7(2):1000229.

[39] Yemane GD . The factors associated with under-five mortality in Ethiopia. Ann Med Surg. 2022;79:104063. 10.1016/j.amsu.2022.104063 PMC9289410 35860052

